# Supplementary material for: Analysis of bacterial genomes from an evolution experiment with horizontal gene transfer shows that recombination can sometimes overwhelm selection
Source: PLoS Genet. 2018 Jan 31;14(1):e1007199. doi: 10.1371/journal.pgen.1007199 (PMC5809092; doi:10.1371/journal.pgen.1007199)
Supplement: S1 Text — (PDF) [file pgen.1007199.s001.pdf]

S1 Text. Calculation of lengths of donor and recipient segments in recombinant genomes.

Each genome is represented by a list of labeled mutations. First, we initialize a list of “0” with the length of the genome. We keep track of two state variables: the index of the last breakpoint (transition-state) and a Boolean state variable called *in.K12.chunk* that is initialized to FALSE under the assumption that the first genomic segment comes from the B recipient. For every labeled mutation in the genome, we check whether the current mutation has a label that changes the state of *in.K12.chunk*. If *in.K12.chunk* is FALSE and its state changes, then the current mutation is labeled “1-2”. If *in.K12.chunk* is TRUE and its state changes, the current mutation is labeled “2-1”. At the end of the loop, we check our initial assumption that *in.K12.chunk* was FALSE. Because we stored the position of the last transition-state, we check whether the last transition-state in the genome is “1-2”, in which case the first “1-2” transition should be set to “0” because the *E. coli* genome is circular. All sites marked “0” are removed from the genome. We then calculate the differences between the N–1 pairs of transition-state mutations: “1-2” on the left and “2-1” on the right gives the length of a K-12 segment, whereas “2-1” on the left and “1-2” on the right gives the length of a B segment. The two final transition-state mutations are the last and first elements of the list. In this way, we calculate the lengths of segments in a recombinant genome that were derived from the donor and recipient, adjusting for any deletions or insertions that may have occurred in those segments.
