# Supplementary figures and images for: Analysis of bacterial genomes from an evolution experiment with horizontal gene transfer shows that recombination can sometimes overwhelm selection
Source: PLoS Genet. 2018 Jan 31;14(1):e1007199. doi: 10.1371/journal.pgen.1007199 (PMC5809092; doi:10.1371/journal.pgen.1007199)

Differences between K-12 and REL606

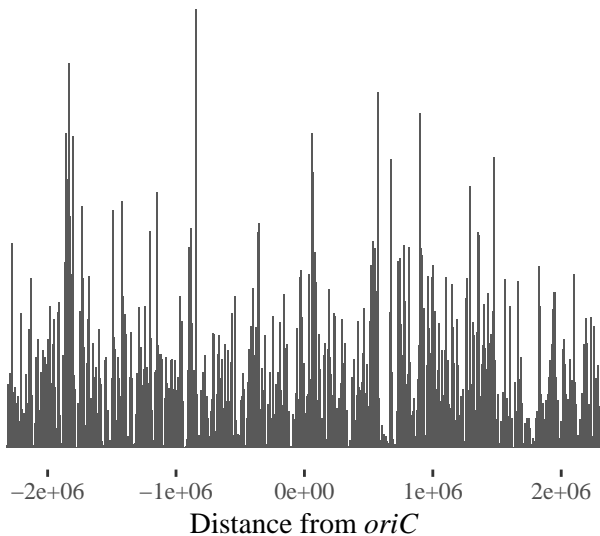

Supplement: S1 Fig — The numbers were binned over 556 DNA segments that are each 8327 bp in length. (PDF) [file pgen.1007199.s004.pdf]

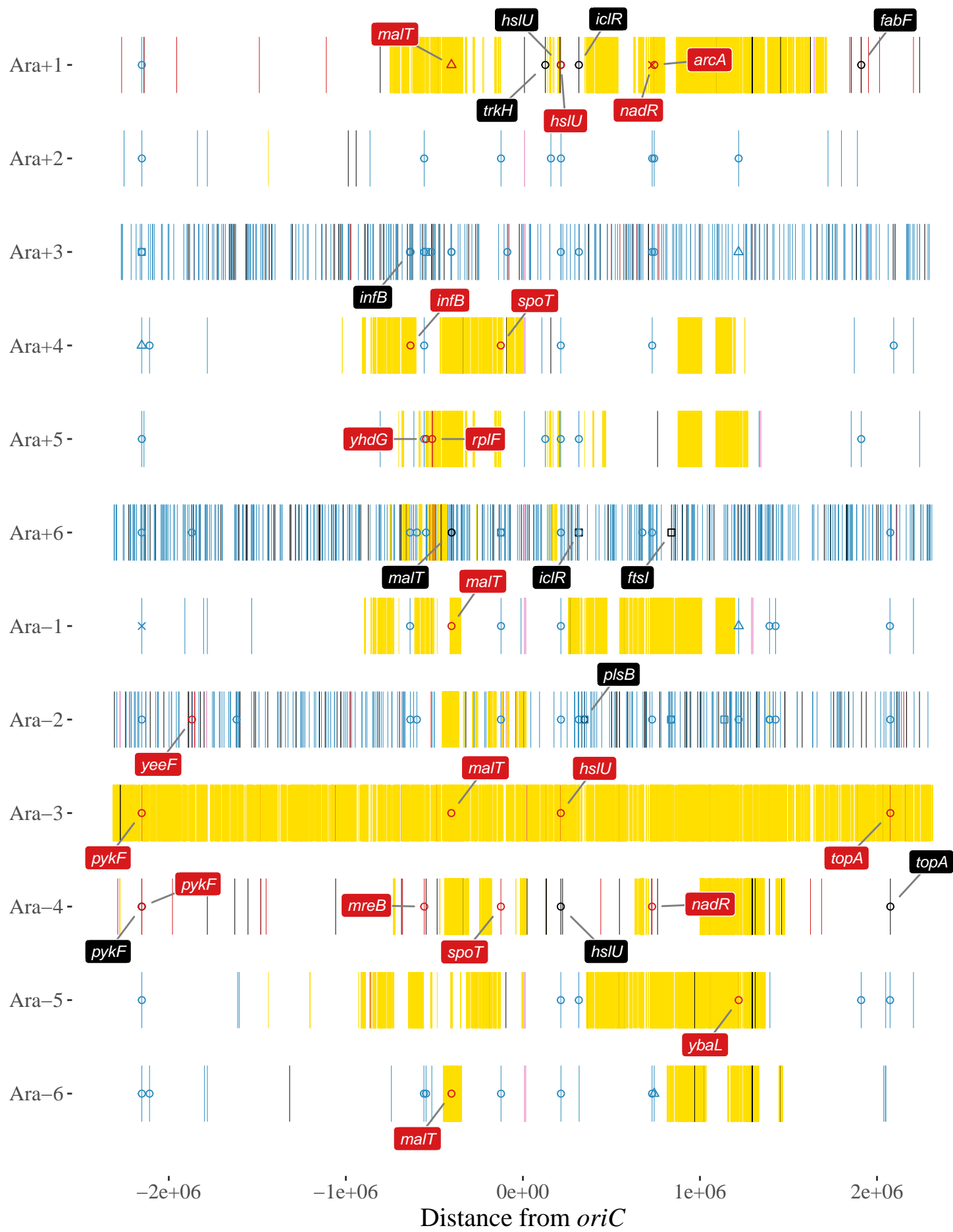

Supplement: S2 Fig — The REL606 genomic coordinates are shown on the x-axis, centered on the oriC origin of replication, and the source populations are shown on the y-axis. Genetic markers are shown as vertical lines, with the color indicating the origin of each marker. Markers specific to K-12 donors are yellow; markers specific to recipient clones are blue; markers in deleted regions are light purple; new mutations that arose during the STLE are black; and LTEE-derived mutations that were replaced by donor DNA during the STLE are red. In addition, symbols indicate mutations in genes under positive selection in the LTEE (Table 1). Open circles indicate nonsynonymous point mutations; open squares are synonymous mutations; open triangles are indels; and x-marks are IS-element insertions. Replaced and new mutations in the genes in Table 1 are labeled by their gene names. (PDF) [file pgen.1007199.s005.pdf]

Ara-3 Clone

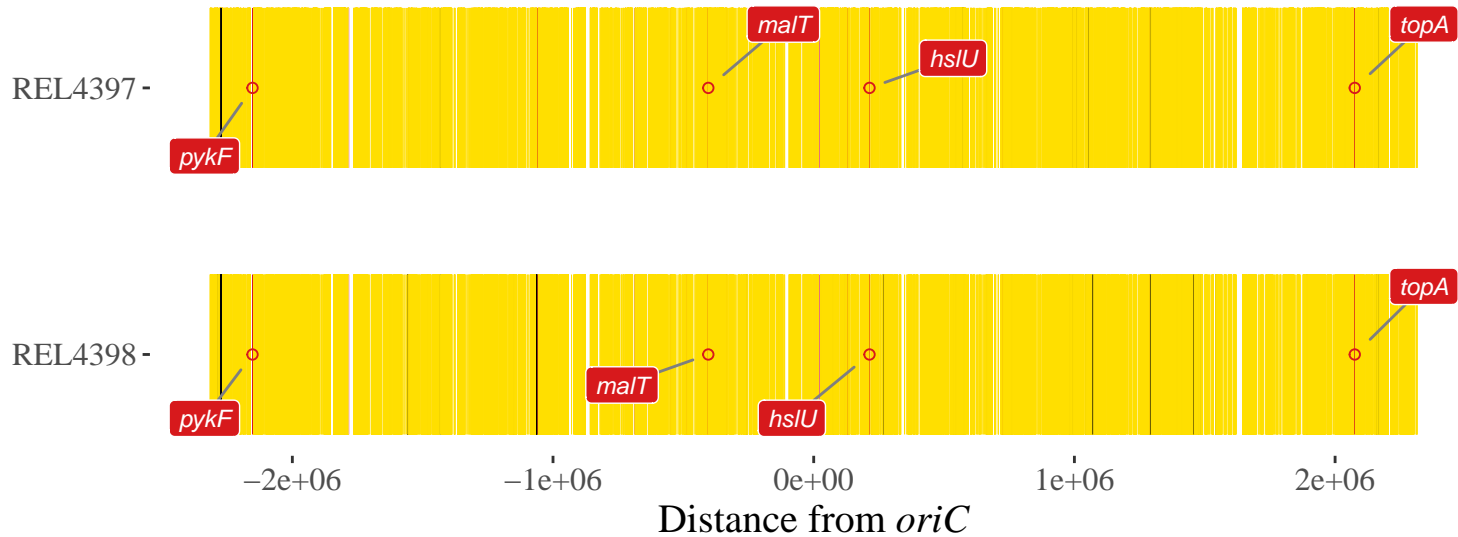

Supplement: S3 Fig — That paper found a cross-feeding interaction between these clones, which puzzlingly had declined in fitness relative to their progenitor during the STLE. The REL606 genomic coordinates are shown on the x-axis, centered on the oriC origin of replication, and the source populations are shown on the y-axis. See legend to S2 Fig for description of symbols and labels. (PDF) [file pgen.1007199.s006.pdf]

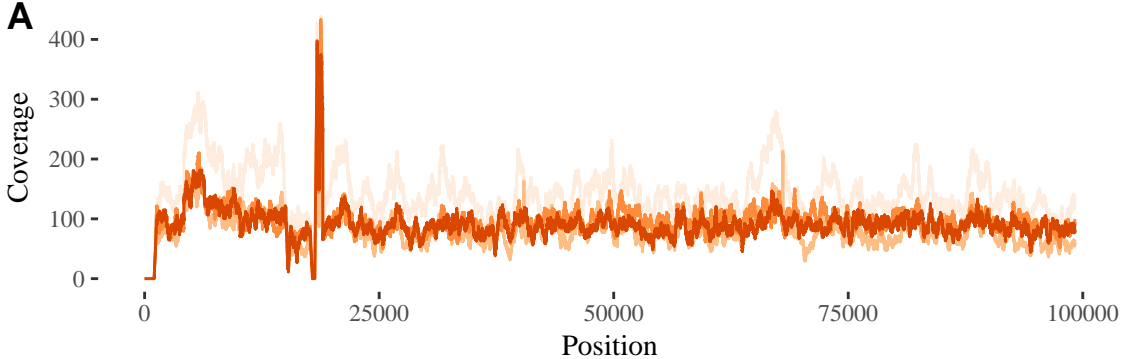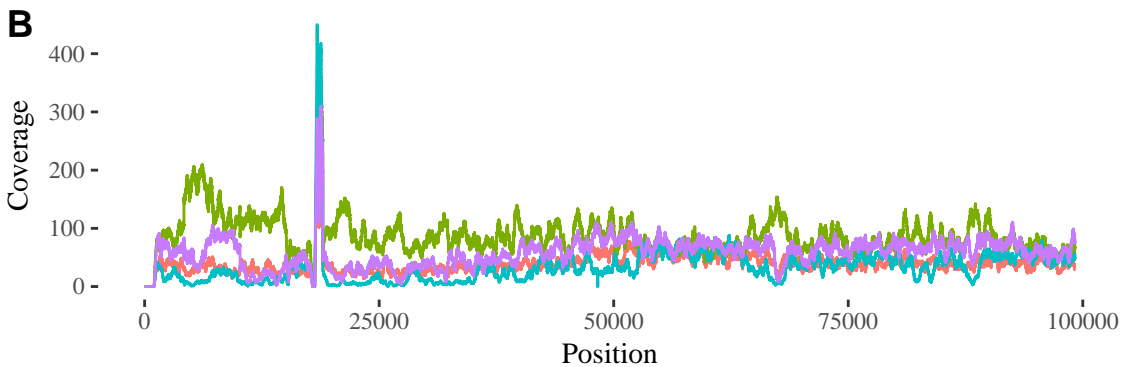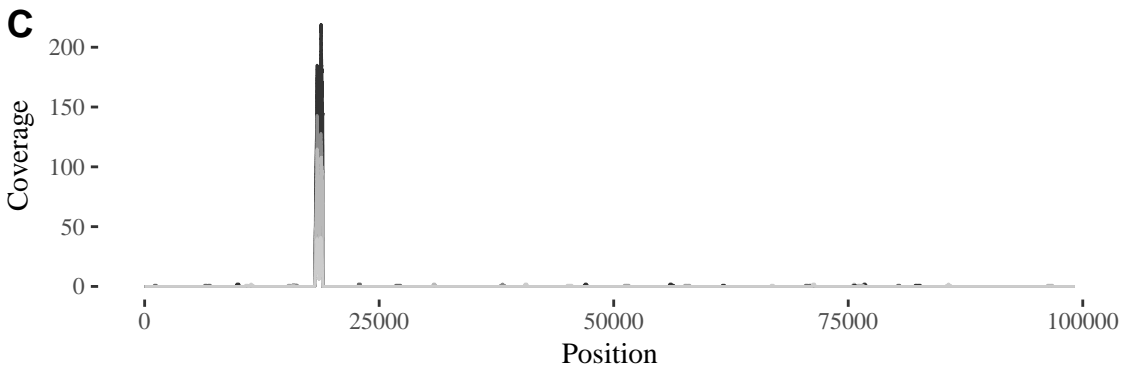

Supplement: S4 Fig — Read coverage over the plasmid reference sequence is plotted for each STLE recombinant clone. The spikes in read coverage near the start and near 20 Kbp of the plasmid reference are false-positive reads that map onto repeat sequences in the plasmid. (A) All Ara–3 STLE clones are shown with REL4397 in very light orange, REL4398 in light orange, REL11750 in medium orange, and REL11751 in dark orange. (B) For comparison, the four Hfr donors are shown with REL288 in red, REL291 green, REL296 teal, and REL298 purple. (C) All other STLE recombinant clones are shown in shades of grey. (PDF) [file pgen.1007199.s007.pdf]

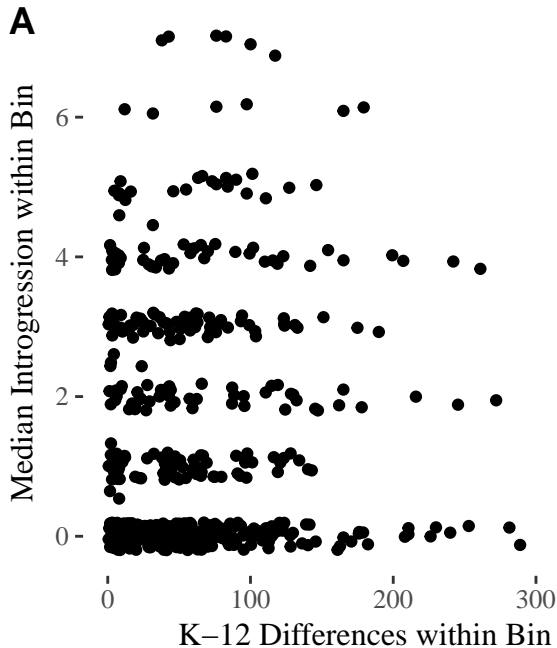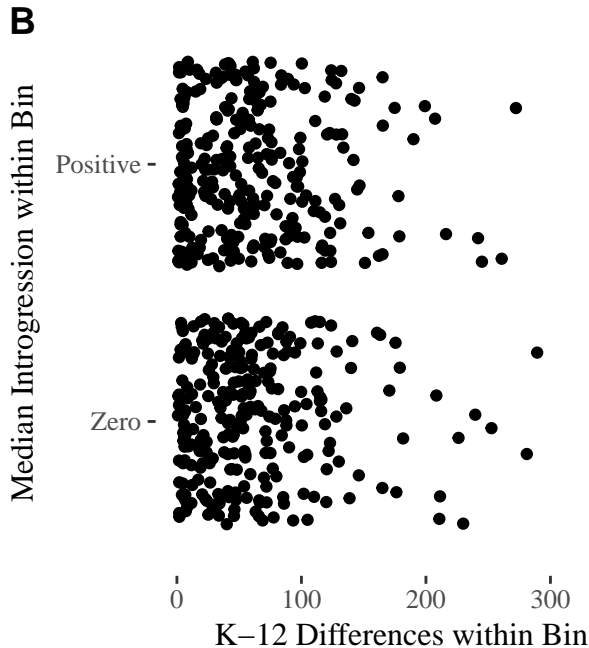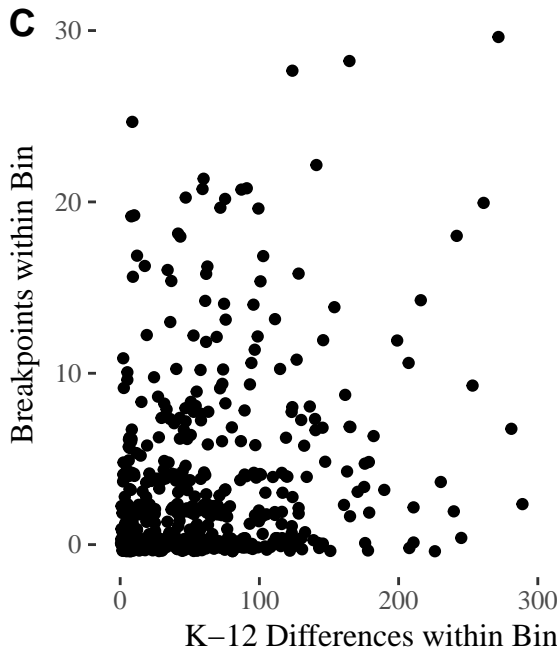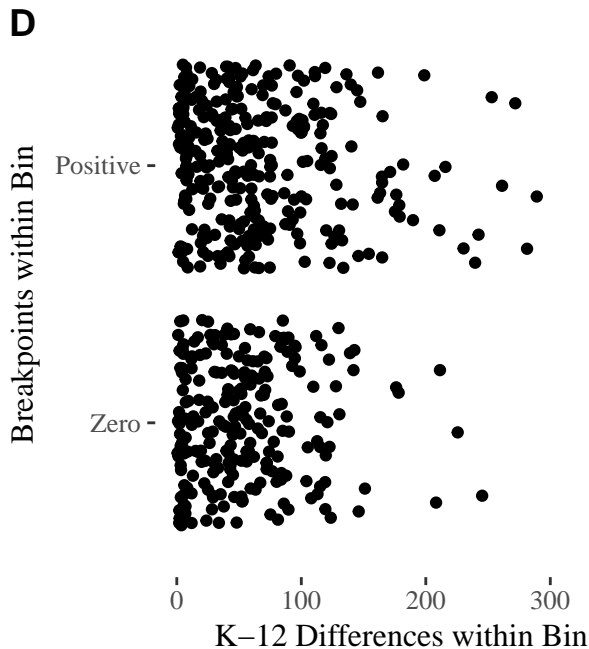

Supplement: S5 Fig — The recipients are all recently derived from REL606, itself a derivative of E. coli B. The REL606 genome is 4,629,812 bp, which we divided into 556 bins that are each 8327 bp long. Each point represents one bin. The x-coordinate for each point shows the number of mutational differences between K-12 and REL606 in the bin. (A) The y-coordinate shows the median number of parallel introgression events (as shown in Fig 2) within that bin, which does not correlate with divergence (Spearman-rank correlation r = 0.0347, P = 0.4183). (B) There is no difference in divergence between bins with and without parallel introgression events (Kruskal-Wallis rank-sum test, P = 0.9235) (C) The y-coordinate shows the number of recombination breakpoints within each bin, which correlates with divergence (Spearman-rank correlation r = 0.1706, P < 0.0001). (D) Bins with breakpoints have diverged more than bins without breakpoints (Kruskal-Wallis rank-sum test, P = 0.0199). (PDF) [file pgen.1007199.s008.pdf]

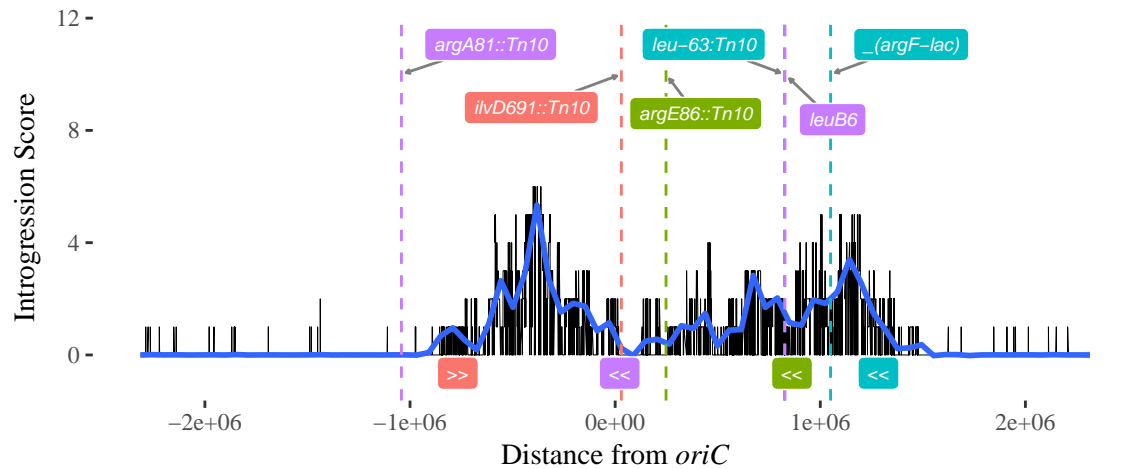

Supplement: S6 Fig — K-12 markers found in both recombinant clones and at 100% frequency in both the initial (1000 generation) and final (1200 generation) samples of the STLE continuation experiment were summed over each population (omitting the Ara–3 population which is almost completely derived from K-12 donor DNA). The locations of auxotroph mutations in the donor genomes are shown as dashed vertical lines, and the location and orientation of the Hfr oriT transfer origin sites are labeled below the x-axis. The four donors are colored as in Fig 2. (PDF) [file pgen.1007199.s009.pdf]

Generation 1000

Generation 1200

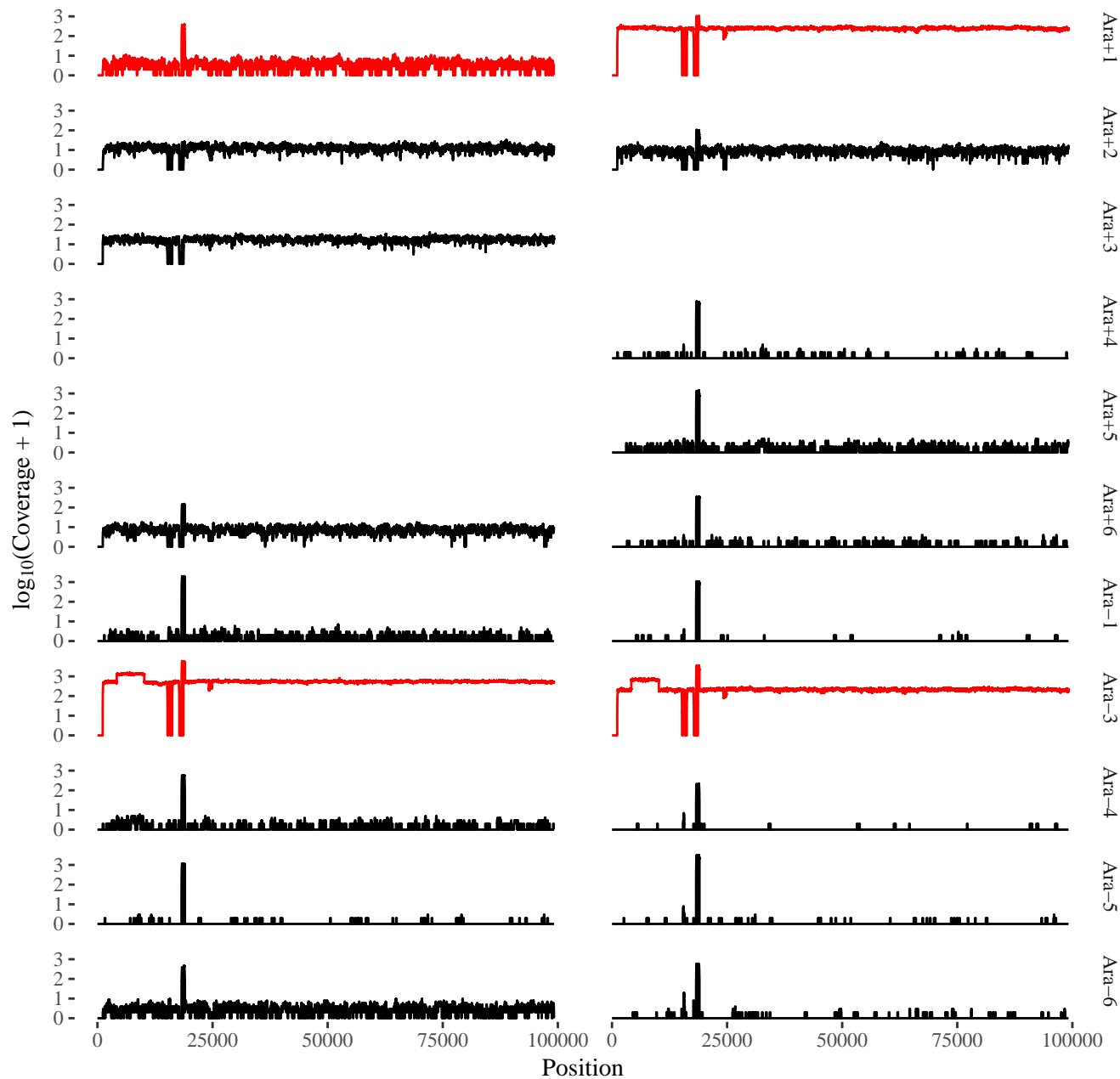

Supplement: S7 Fig — The Ara+1 and Ara–3 populations are shown in red, and all other populations in black. This plot shows the coverage distribution over the plasmid reference sequence for the initial (generation 1000) and final (generation 1200) samples from the continuation populations. The three blank panels are for samples where breseq (version 0.31) indicated insufficient coverage across the F plasmid. (PDF) [file pgen.1007199.s010.pdf]
